# In silico Identification of Hypoxic Signature followed by reverse transcription-quantitative PCR Validation in Cancer Cell Lines

**DOI:** 10.52547/ibj.3803

**Published:** 2022-12-12

**Authors:** Sara Shayan, Golnaz Bahramali, Arash Arashkia, Kayhan Azadmanesh

**Affiliations:** 1 Department of Molecular Virology, Pasteur Institute of Iran, Tehran, Iran;; 2 Department of Hepatitis and AIDS and Blood Borne Diseases, Pasteur Institute of Iran, Tehran, Iran

**Keywords:** Hypoxia, MicroRNA, RNAseq

## Abstract

**Background:**

Hypoxic tumor microenvironment is one of the important impediments for conventional cancer therapy. This study aimed to computationally identify hypoxia-related mRNA signatures in nine hypoxic-conditioned cancer cell lines and investigate their role during hypoxia.

**Methods:**

Nine RNA-Seq expression data sets were retrieved from the Gene Expression Omnibus database. DEGs were identified in each cancer cell line. Then 23 common DEGs were selected by comparing the gene lists across the nine cancer cell lines. qRT-PCR was performed to validate the identified DEGs.

**Results:**

By comparing the data sets, GAPDH, LRP1, ALDOA, EFEMP2, PLOD2, CA9, EGLN3, HK, PDK1, KDM3A, UBC, and P4HA1 were identified as hub genes. In addition, miR-335-5p, miR-122-5p, miR-6807-5p, miR-1915-3p, miR-6764-5p, miR-92-3p, miR-23b-3p, miR-615-3p, miR-124-3p, miR-484, and miR-455-3p were determined as common miRNAs. Four DEGs were selected for mRNA expression validation in cancer cells under normoxic and hypoxic conditions with qRT-PCR. The results also showed that the expression levels determined by qRT-PCR were consistent with RNA-Seq data.

**Conclusion:**

The identified PPI network of common DEGs could serve as potential hypoxia biomarkers and might be helpful for improving therapeutic strategies.

## INTRODUCTION

A gene expression signature is a single or combined group of genes whose expression responds to a particular signal or changes in cellular status in a predictable way. Gene signatures are frequently extracted from a set of DEGs by comparing two groups, such as cell lines under different treatment conditions. Gene expression signatures can therefore be used as surrogate markers to comprehend the complexity of pathway activation.

Oxygen deprivation occurs in almost all solid tumors. A shortage of oxygen is the consequence of inadequate oxygen delivery via inefficient tumor vasculature^[^^1^^]^. Hypoxia affects tumor behavior and facilitates tumor progression and metastasis, leading to resistance to conventional chemo- and radiotherapy^[^^2^^]^. Therefore, identifying the key genes regulating cancer cell behavior during hypoxia is essential for developing anticancer agents that efficiently kill tumor cells under hypoxic conditions.

A growing number of studies have identified DEGs during hypoxia in different cancer cell types using RNA-Seq analysis^[^^3^^-^^5^^]^. However, their findings only represent the genetic characteristics of specific tumor cells during hypoxia. In this study, we used RNA-Seq datasets of nine different hypoxic-conditioned cancer cell lines to find hypoxia-related mRNA signatures. Since human cancer cell lines are widely used for better understanding of cancer biology, cancer cell characterization, and anticancer drug discovery^[^^6^^]^, we selected the available RNA-Seq datasets of cell lines to explore the effect of hypoxia on gene expression profiles. 

MiRNAs play a central role in regulating gene expression^[^^7^^]^. Kulshreshtha and colleagues^[^^8^^]^ described a functional link between hypoxia and miRNA expression. They indicated that miRNAs profile are regulated by hypoxia in a variety of cell types, and their dysregulation is associated with many cancers, making their signature a potential prognostic biomarker^[^^9^^]^. In the present study, common DEGs along with their hub genes among the nine different cancer cell lines were screened during hypoxia. Then we investigated a PPI network and predicted a miRNA-targeted gene network, which might provide a basis for further studies. Our aim was to discover the molecular mechanism underlying the effect of hypoxia and provide potential prognostic markers.

## MATERIALS AND METHODS

**Raw biological data and differential RNA expression analysis**

Raw RNA-Seq data of nine hypoxia-conditioned cancer cell lines were retrieved from the Sequence Read Archive (www.ncbi.nlm.nih.gov/geo). Among these datasets, GSE131378 contained four samples of hypoxic-conditioned and four samples of normoxic-conditioned A549 cells, while GSE72437 consisted of five samples of hypoxic-conditioned and five samples of normoxic-conditioned BeWo cells. Moreover, GSE78025, GSE81513, GSE84167, GSE13967, GSE149132, and GSE160491 contained three samples of hypoxic-conditioned and also three samples of normoxic-conditioned U78-MG, HCT116, MCF-7, ASPC-1, T47D, and BCPAP, respectively. GSE131379 also comprised of two samples of hypoxic-conditioned and three samples of normoxic-conditioned Hela cells. SAMtools was used to extract raw sequencing reads. The read quality was examined using FastQC version 0.11.2, and low quality bases and adaptor sequences were removed using Trimmomatic version 0.32; the expression level of each transcript was then quantified in transcripts per million using Kallisto^[10]^. The counts were imported into software R v. 3.4.0 using the tximport R package v. 1.4.0, and the DEGs were identified with a | log2 fold change | ≥1 and a false discovery rate <0.05 using the DESeq2 package in R v. 3.2.3. The UpSetR package in R was employed to ﬁnd common genes between diﬀerent datasets^[11]^. The default values were employed for all the packages.

**Function enrichment analysis**

We used Database for Annotation, Visualization, and Integrated discovery (DAVID) (https://david.ncifcrf. gov/; version 6.8) for GO functional analysis and KEGG pathway analysis of DEGs^[^^12^^-^^14^^]^. The Evolutionary Relationships (PANTHER) was also used to determine protein class over-representation^[^^15^^]^, and *p* < 0.05 represented statistical significance. 

**Construction of a** **PPI network **

Interactions between the common DEGs and other proteins would be useful to fully understand their biological roles. In this study, 23 common DEG PPI network were constructed by Retrieval of Interacting Genes (STRING; https://string-db.org/). Moreover, 23 common DEGs were integrated into the International Molecular Exchange Consortium database (https:// www.imexconsortium.org/) to identify the hub genes information in PPI network^[^^16^^]^. The protein interaction network was visualized using NetworkAnalyst (https://www.networkanalyst.ca) and Cytoscape (3.9.1)^[^^16^^]^. To evaluate the nodes in the PPI network, we adopted several topological measures, including degree (*k*), MCC, BC, and CC. Since degree (*k*), BC, and MCC are often used for detecting the hub in a network^[^^17^^-^^19^^]^, we determined hub genes based on connectivity degree (number of interactions) >10, MCC, and BC using Cytohubba on Cytoscape. 

**MiRNA interactions analysis**

To identify the miRNA-mRNA target interactions, miRTarBase^[^^20^^]^ and TarBase^[^^21^^]^ (both version 8.0) were employed to collect the miRNA-gene interaction data. Topological analysis based on degree and betweenness centrality as key topological parameters was performed utilizing NetworkAnalyst. 

**Cell culture for qRT-PCR validation**

To validate our findings, we selected four hub genes, including *GAPDH*, *LRP1*, *ALDOA*, and *PLOD2* to determine their expression in cancer cell lines (A549, U78-MG, HCT116, Hela, and MCF-7) under hypoxic or normoxic conditions. Cells were purchased from the National Cell Bank of Iran (Pasteur Institute, Tehran, Iran). Cells used in the experiment were cultured in DMEM supplemented with 10% FBS and incubated in a humidified incubator with 5% CO2 at 37 °C.

**Cancer cell adaptation to hypoxia**

Cells were seeded in a T25 flask and cultured in DMEM medium supplemented with 10% FBS. The cells were repeatedly incubated in hypoxic conditions in an Anoxomat chamber (Mart Microbiology, Lichtenvoorde, The Netherland; 1% O_2_) for 4 h and then incubated in a standard culture environment (5% CO_2_ and 95% air) at 37 °C for 48-72 h. Cells were treated twice weekly, and hypoxic-conditioned cell lines were generated after 20 exposures to hypoxia^[^^22^^]^. 

**RNA isolation and qRT-PCR**

Trizol reagent (TaKara, Kusatsu, Shiga, Japan) was used for RNA isolation from the cells during normoxia and hypoxia. RNA samples were reversely transcribed to complementary DNA by the QIAGEN Reverse Transcription Kit (Qiagen, Germany). Subsequently, the quantification of cDNA was performed by the qRT-PCR method using SYBR Green Master Mix (Amplicon). The reaction conditions were conducted at 95 °C for 10 min, 40 cycles of 95 °C for 10 s, 60 °C for 30 s, and 72 °C for 30 s. The *RPLP0* was used as an internal reference control^[^^23^^]^. Gene expression levels were calculated based on the Delta-Delta Ct relative quantification. 

**Statistical analysis**

Statistical analyses were performed using the student’s t-test with GraphPad Prism 8 software (GraphPad Prism, San Diego, CA, USA). The *p* value was considered statistically significant when it was less than 0.05.

## RESULTS

**Differential RNA expression analysis**

**Fig. 1:**
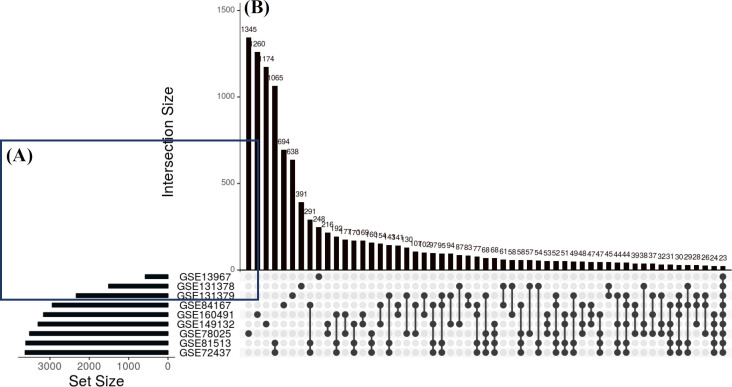
UpSet plot of DEGs. (A) Total number of DEGs during hypoxia; (B) intersection of gene sets in hypoxic conditions. Black circles indicate the total number of DEGs with differences in log2 fold change expression in each dataset, and connecting bars show the overlapping DEGs

RNA sequencing data from the nine different hypoxic-conditioned cancer cell lines (A549, BeWo, U78-MG, HCT116, Hela, MCF-7, ASPC-1, T47D, and BCPAP) were analyzed, and 23 common DEGs were identified (Fig. 1), including *EGLN3*,* ANGPTL4*,* GPR146*,* C4orf47*,* KCTD11*,* CA9*,* PPFIA4*,* PLOD2*,* HK2*, and *TMEM*. Interestingly, all of these genes were upregulated in the hypoxic-conditioned cancer cell lines.

**Functional categories and pathway analysis**

The PANTHER protein classification revealed that the common DEGs were classified into nine groups according to their function: protein modifying enzyme (*PPFIA4*, *PDK1*, and *PLOD2*), scaffold/adaptor protein (*KCTD11*), transfer/carrier protein (*LRP1*), transmembrane signal receptor (*GPR146*), cytoskeletal protein (*HK2*), extracellular matrix protein (*EFEMP2*), intercellular signal molecule (*ANGPTL4*), metabolite interconversion enzyme (*FUT11*, *GAPDH*, *QSOX1*, *PFKFB4*, *ALDOA*, and *HK2*), and regulatory protein (*KDM3A*). GO analysis, which covered the three GO categories (i.e. CC, BP, and MF), was performed using DAVID. DEGS were enriched significantly in different GO terms, including hexose metabolic process (ontology: BP), monosaccharide binding (ontology: MF), and mitochondrial pyruvate dehydrogenase complex (ontology: CCO); the results are summarized in Table 1. The significance threshold of *p *< 0.05 was selected. Moreover, seven pathways were significantly enriched based on KEGG pathway analysis, including HIF-1 signaling pathway, fructose and mannose metabolism, glycolysis/gluconeogenesis, carbon metabolism, cholesterol metabolism, central carbon metabolism in cancer, and biosynthesis of amino acids (Table 2).

**PPI network construction and hub gene selection**

**Fig. 2:**
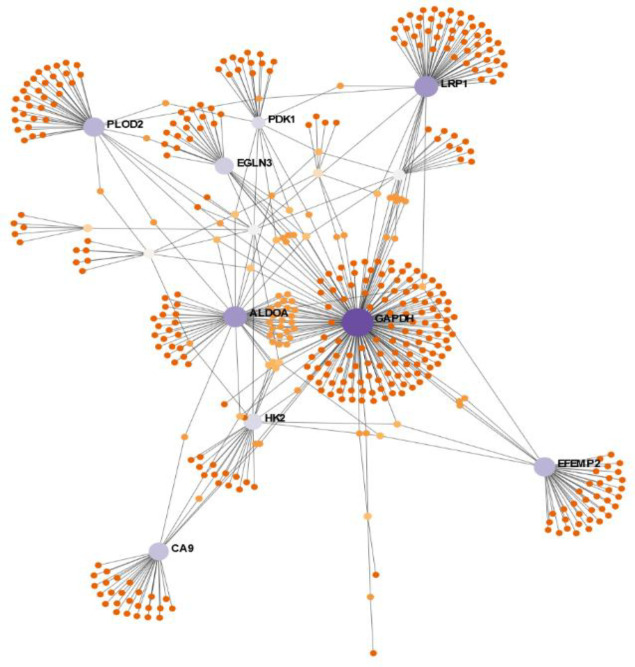
PPI network of common genes among nine different cell lines during hypoxia by mapping DEGs into the NetworkAnalyst database. Purple nodes represent the 23 common DEGs, and the area of each circle demonstrates the degree of the node in the network. The color of nodes is proportional to their BC values

**Fig. 3:**
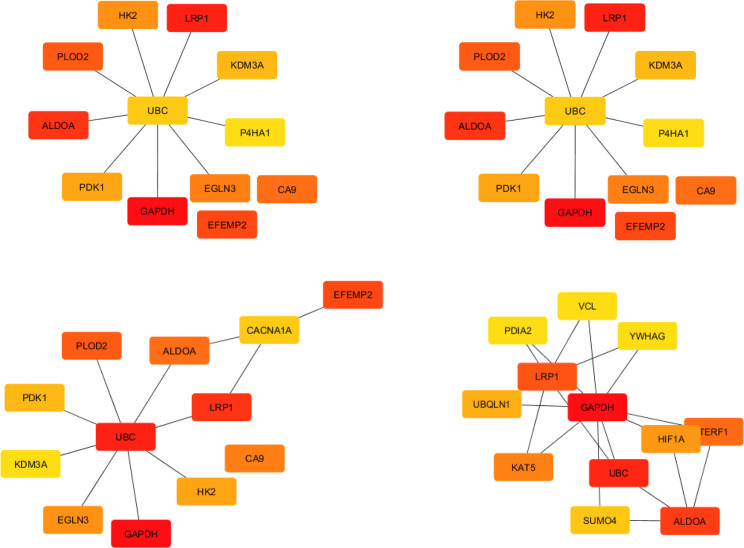
Results of algorithms from the Cytohubba. Hub genes were screened by degree, MCC, and BC according to the Cytohubba plug-in. Centrality in the network was measured by CC. The more forward ranking is represented by a redder color

 Using the STRING database, a PPI network obtained from 23 common DEGs, which was composed of 22 nodes and 25 edges, was constructed and visualized in Cytoscape (Supplementary Fig. 1). In order to screen the PPI network’s interactions with other proteins, which provide important clues about their functions, the PPI network was integrated into the International Molecular Exchange Consortium database. A PPI network composed of 448 nodes and 531 edges was obtained (Fig. 2). Twelve hub proteins, including *GAPDH*,* LRP1*,* ALDOA*,* EFEMP2*,* PLOD2*,* CA9*,* EGLN3*,* HK*,* PDK1*,* KDM3A*,* UBC*, and *P4HA1*, were identified in this network based on degrees (>10), MCC, and BC (Fig. 3 and Table 3). 

**Gene regulatory network analysis**

**Fig. 4:**
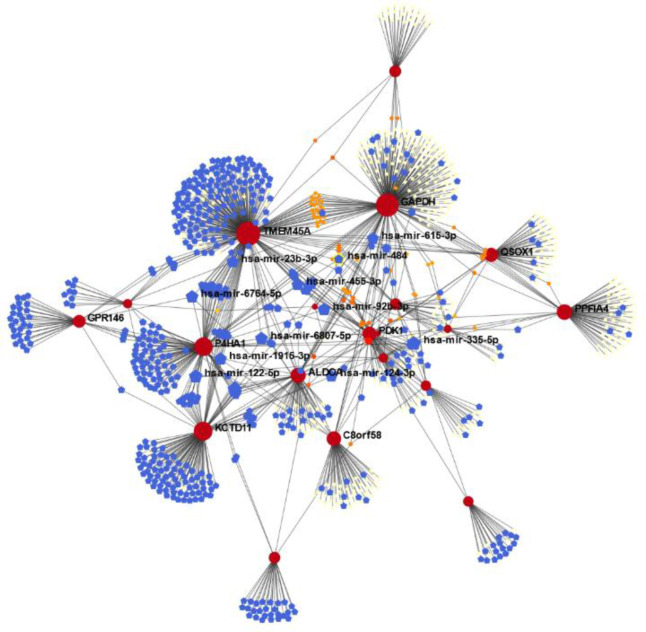
Network analysis of DEG-miRNA interactions. NetworkAnalyst was used to visualize data obtained from the miRTarBase and TarBase databases. Blue squares represent microRNAs, and red circles represent genes. The area of each circle demonstrates the degree of the node in the network. The color of nodes is proportional to their BC values

The key miRNAs (miR-335-5p, miR-122-5p, miRr-6807-5p, miR-1915-3p, miR-6764-5p, mirR-92-3p, miR-23b-3p, miR-615-3p, miR-124-3p, miR-484, and miR-455-3p) were identified based on network topological properties (degree and betweenness centrality). Additionally, our results indicate miR-92-3p can regulate a large number of mRNA targets (n = 88), as shown by the PPI network (Fig. 4).

**Table 1:** Top 10 GO analyses of the diﬀerentially expressed genes identiﬁed from RNA-Seq data of hypoxic-conditioned cell lines

**Category**	**GO ID**	**Term**	***p*** ** value**
Biological process	0019318	Hexose metabolic process	0.00067
	0001666	Response to hypoxia	0.0011
	0006006	Glucose metabolic process	0.0011
	0006735	NADH regeneration	0.0011
	0009435	NAD biosynthetic process	0.0011
	0018126	Protein hydroxylation	0.0011
	0018401	Peptidyl-proline hydroxylation to 4-hydroxy-L-proline	0.0011
	0042866	Pyruvate biosynthetic process	0.0011
	0055114	Oxidation-reduction process	0.0011
	0061621	Canonical glycolysis	0.0011
			
Molecular function	0048029	Monosaccharide binding	2.18E-06
	0031418	L-ascorbic acid binding	0.00017
	0051213	Dioxygenase activity	0.00017
	0005506	Iron ion binding	0.00062
	0016706	2-oxoglutarate-dependent dioxygenase activity	0.00062
	0031545	Peptidyl-proline 4-dioxygenase activity	0.00099
	0016491	Ooxidoreductase activity	0.002
	0050662	Coenzyme binding	0.0038
	0019200	Carbohydrate kinase activity	0.0044
	0048037	Cofactor binding	0.0191
			
Cellular component	0005967	Mitochondrial pyruvate dehydrogenase complex	0.00581
	1990204	Oxidoreductase	0.00747
	0070820	Tertiary granule	0.0158
	0016323	Basolateral plasma membrane	0.0289
	0009925	Basal plasma membrane	0.0349
	0045178	Basal part of cell	0.0398
	0005813	Centrosome	0.0348
	0099512	Supramolecular fibre	0.0275
	0099081	Supramolecular polymer	0.0282
	0005856	Cytoskeleton	0.0482

*p * < 0.05 considered significant

**Table 2:** The KEGG pathway analysis of the overlapping DEGs associated with hypoxia

**Category**	**Pathways**	**count**	***p*** ** value**
KEGG	HIF-1 signalling pathway	5	2.41E-06
	Fructose and mannose metabolism	3	0.00012
	Glycolysis/gluconeogenesis	3	0.0006
	Carbon metabolism	3	0.0021
	Cholesterol metabolism	2	0.0077
	Central carbon metabolism in cancer	2	0.0115
	Biosynthesis of amino acids	2	0.0119

**Quantitative real-time PCR for DEGs**

**Fig. 5:**
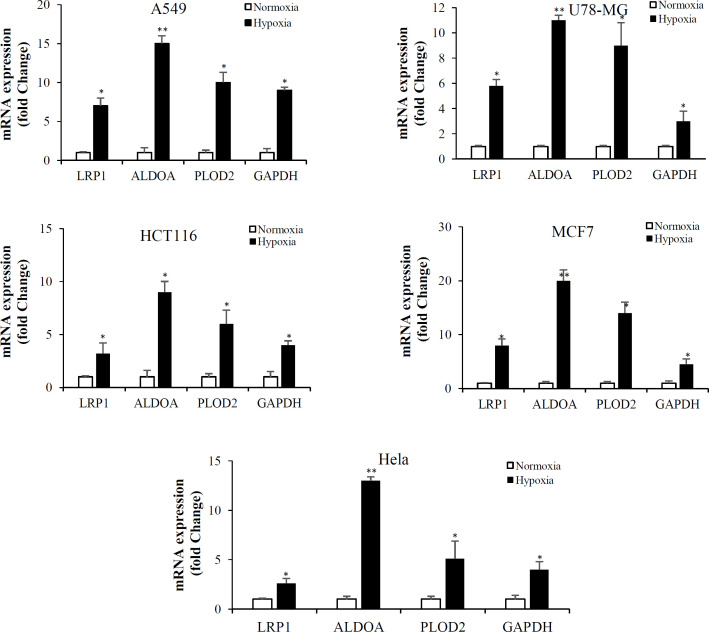
The mRNA expression of GAPDH, LRP1, ALDOA, and PLOD2 in A549, U78-MG, HCT116, MCF-7, and Hela cells under normoxic and hypoxic conditions analyzed by qRT-PCR. Gene expression levels were calculated based on Delta-delta Ct relative quantification. The data represents at least two biological replicates, each of which was run in triplicate (^*^p < 0.05; ^**^p < 0.01)

 In order to validate the DEGs identified by RNA-seq analysis, four hub genes, including GAPDH, LRP1, ALDOA, and PLOD2, were selected for analysis via qRT-PCR under normoxic and hypoxic conditions. Primers were designed based on available sequences to amplify the specific altered genes. Primer sequences are shown in Table 4. Based on the qRT-PCR results, the candidate genes were upregulated in A549, U78-MG, HCT116, Hela, and MCF-7 cells under hypoxic conditions (Fig. 5). The expression profiles of four genes confirmed the original transcriptome data obtained by RNA-Seq. 

## DISCUSSION

Because hypoxic cells are likely to be resistant to chemo- and radiotherapy, it is of high importance to identify the key hypoxia-inducible genes and resistance mechanisms for efficient therapeutic intervention. Moreover, it is well established that miRNA plays a central role in regulating the various biological pathways^[^^24^^]^. Therefore, exploring the role and impact of mRNA and miRNA in cancer cells, especially during hypoxia, could be helpful in cancer diagnosis and treatment. 

In the current study, we conducted bioinformatics analysis to identify the candidate key genes and biological pathways among nine different cancer cell lines exposed to hypoxic conditions. Data was extracted from GSE131378, GSE72437, GSE78025, GSE81513, GSE131379, GSE84167, GSE13967, GSE149132, and GSE160491 datasets, among which 23 common DEGs were screened. To our surprise, all the common DEGs were upregulated in all the nine hypoxic-conditioned cancer cell lines. In order to gain some insight into how hypoxia affects the expression of genes at the molecular level, GO and KEGG pathway enrichment analyses were carried out^[^^13^^,^^14^^]^. Functional enrichment analysis revealed that the hexose metabolic process, response to hypoxia, and glucose metabolic process were significantly changed. According to KEGG enrichment analysis, 23 common genes were enriched in the *HIF*-*1* signaling pathway, including fructose and mannose metabolism, glycolysis/gluconeogenesis, carbon metabolism, cholesterol metabolism, central carbon metabolism in cancer, and biosynthesis of amino acids. Since it is believed that proteins with more interactions have higher chances of being involved in the essential PPI^[^^25^^]^, the PPI network was constructed and *GAPDH, LRP1, ALDOA, EFEMP2, PLOD2, CA9, EGLN3, HK*, and *PDK1* were identified as the hub genes. 

To support our findings, we selected four hub genes (*GAPDH, LRP1, ALDOA, and PLOD2*) for qRT-PCR validation in A549, U78-MG, HCT116, Hela, and MCF-7 cells under normoxic and hypoxic conditions. Expression patterns of four genes generated by qRT-PCR were consistent with RNA-seq data. Consistently, several studies have found that hypoxia-related genes such as *GAPDH, LRP1, ALDOA, EFEMP2, PLOD2, CA9, EGLN3, HK*, and *PDK1* are upregulated during hypoxia^[^^26^^-^^28^^]^.

**Table 3:** Summary of the selected hub proteins based on degree, MCC, and BC in hypoxic-conditioned cell lines (A549, BeWo, U78-MG, HCT116, Hela, MCF-7, ASPC-1, T47D, and BCPA)

**Symbol**	**Description**	**Degree**	**BC**	**CC**
GAPDH	Glyceraldehyde3-phosphate dehydrogenase	183	66486.07	273.75
LRP1	Low density lipoprotein receptor-related protein 1	59	21784.76	186.65
ALDOA	Aldolase A	57	15174.48	189.15
EFEMP2	EGF containing fibulin extracellular matrix protein 2	41	14612.74	160.2667
PLOD2	Procollagen-lysine,2-oxoglutarate 5-dioxygenase 2	40	14259.61	168.2833
CA9	Carbonic anhydrase 9	30	10950.54	144.0333
EGLN3	Egl nine homolog 3	25	7895	158.2833
HK2	Hexokinase 2	22	7509.14	164.7833
PDK1	Pyruvate dehydrogenase kinase 1	21	6557.02	156.65
KDM3A	Lysine demethylase 3A	15	4690.6	151.8667
UBC	Ubiquitin C	13	22879.18	212.3667
P4HA1	Prolyl 4-Hydroxylase Subunit Alpha 1	11	3109.74	149.9833

Since there is no edge between the neighbors of the node, the MCC is equal to its degree.

*GAPDH* and *ALDOA* are involved in glycolysis. It is widely believed that the overexpression of glycolytic enzymes in a large number of tumors compensates for the increased energy demands and supports rapid tumor growth^[^^29^^]^. However, many glycolytic enzymes have non-glycolytic functions, as well^[^^30^^]^. For instance, overexpressed *GAPDH* could inhibit caspase-independent cell death by inducing Bcl-xL upregulation, leading to cancer cell survival and resistance to chemotherapeutic agents^[^^31^^,^^32^^]^. Moreover, *GAPDH* protects cancer cells against chemotherapy by directly binding to the telomeric DNA and prevents the rapid degradation of telomeres^[^^33^^]^. More importantly, *GAPDH*, which is perceived as a common reference gene, is upregulates under hypoxic conditions. Therefore, using *GAPDH* as a housekeeping gene should be avoided due to its unstable expression level during hypoxia. 

*ALDOA* and *PDK1* are glycolytic enzymes that contribute to the progress of cancer and metastasis^[^^34^^-^^37^^]^. *ALDOA* overexpression could suppress the expression of proteins responsible for cell-cell adhesion and induce the expression of epithelial-mesenchymal transition^[^^34^^]^. Chang et al.^[^^34^^]^ have demonstrated a feedback loop between *ALDOA* and *HIF*-1, by which *ALDOA* activates *HIF-1α/MMP9* and promotes cancer cell invasion. Under hypoxic conditions, *PDK1* attenuates mitochondrial respiration and ROS production by inactivating the pyruvate dehydrogenase^[^^38^^]^. Additionally, Gibadulinova et al.^[39]^ have indicated that carbonic anhydrase IX promotes metabolic adaptation to hypoxia through the regulation of *PDK1*. A number of studies have also revealed that *PDK1* overexpression promotes cancer cell metastasis, but the molecular mechanism is unclear^[^^36^^,^^37^^]^. Siu et al.^[^^37^^] ^have explained that *PDK1* expression is associated with ovarian cancer metastasis through the activation of *JNK/IL-8* signaling. It has also been displayed that procollagen-lysine, 2-oxoglutarate, *PLOD2* promotes migration and invasion of cancer cells during hypoxia. *PLOD2*, a regulator of collagen cross-linking, is located in the upstream of HK2 and can regulate *HK2* expression through the activation of signal transducer and activator of transcription 3 (STAT3)^[^^40^^]^. 

To predict the correlation of common DEGs with miRNA, a DEG-miRNA network was constructed (Fig. 3). These miRNAs have been reported in some cancer types. We also identified miR-335-5p, miR-122-5p, miR-6807-5p, miR-1915-3p, miR-6764-5p, miR-92-3p, miR-23b-3p, miR-615-3p, miR-124-3p, miR-484, and miR-455-3p as the key interacting miRNAs in hypoxia in different cancer cell lines. The miR-335-5p has been exhibited to have ability to regulate cancer cell metastasis. Zhang et al.^[^^41^^]^ showed that miR-335-5p can promote apoptosis in prostate cancer cells and may be used as a biomarker in the treatment of this disease^[^^41^^,^^42^^]^. Upregulation of miR-6807-5p was reported in glioma specimens^[^^43^^]^. Dysregulation of miR-6764-5p was also identified in pituitary adenomas^[^^44^^]^. MiR-92-3p and miR-122-5p have been identified as the markers of hypoxic environments. MiR-92-3p can be used as a potential therapeutic target in patients with metastatic colorectal cancer^[^^45^^,^^46^^]^. MiR-455-5p is dysregulated in many tumor cells^[^^47^^,^^48^^]^, while miR-1915-3p and miR-124-3p could inhibit apoptosis, resulting in cancer progression. It has been exhibited that miR-1915-3p may play a role in the progression of gastric cancer and may have a potential therapeutic application in gastric cancer^[^^49^^,^^50^^]^. Contradictorily, miR-484 could promote apoptosis by targeting Apaf-1^[^^51^^]^, and miR-23b-3p and miR-615-3p could act as either tumor suppressors or oncogenes, which mainly depends on their context^[^^52^^,^^53^^]^.

**Table 4:** PCR primers used for the validation of gene expression by qRT-PCR

**Gene-specific primers**	**Oligonucleotide primer sequence 5' to 3'**
RPLP0	F: CCATTCTATCATCAACGGGTACAA
	R: TCAGCAAGTGGGAAGGTGTAATC
	
GAPDH	F: GCCATCAATGACCCCTTCAT
	R: GCCATGGAATTTGCCAT
	
LRP1	F: CAACGGCATCTCAGTGGACTAC
	R: TGTTGCTGGACAGAACCACCTC
	
ALDOA	F: GACACTCTACCAGAAGGCGGAT
	R: GGTGGTAGTCTCGCCATTTGTC
	
PLOD2	F: GACAGCGTTCTCTTCGTCCTCA
	R: CTCCAGCCTTTTCGTGGTGACT

In summary, the present study identified hypoxia-related gene signatures among the hypoxia-conditioned cancer cell lines using RNA-Seq. Our analysis revealed the common hub genes and key pathways in cancer cells under hypoxic conditions. Moreover, we predicted a miRNA signature, among which miR-335-5p had the highest betweenness centrality during hypoxia. To our knowledge, for the first time, our results demonstrate that miR-6807-5p and miR-6764-5p are dysregulated under hypoxic conditions. However, further molecular biological experiments are required to confirm the function of the identified miRNA associated with hypoxia. The results of the present study may provide future directions in identifying the presence of cancer and determining the characteristics of cancer. For instance, hypoxia is a characteristic feature of cancer, and the hypoxia signature identified in this study, as well as predicted miRNAs might be helpful to detect the hypoxic state of cancer cells. Hypoxia is common in majority of malignant tumors and an attractive therapeutic target. As hypoxia targeted treatment are effective in patients with the most hypoxic tumors, hypoxic signature might be useful for developing proper treatment, such as engineered oncolytic viruses that could be utilized to control or regulate the biological interactions responsible for the functioning or malfunctioning of cancer cells during hypoxia^[^^22^^]^.

## DECLARATIONS

### Ethical statement

Not applicable. 

### Data availability

The raw data supporting the conclusions of this article are available from the authors upon request.

### Author contributions

SS: conceived, designed the analysis and performed the analysis; GB: performed bioinformatics analyses; AA: drafted or provided critical revision of the article; KA: conceived and designed the study, supervised the data analysis and interpretation. All authors have read and approved the final manuscript.

### Conflict of interest

None declared.

### Funding/support

This study was funded as Ph.D. student project by Pasteur Institute of Iran, Tehran.

## Supplementary Materials

Supplementary Fig. 1
