# Supplementary material for: In silico Identification of Hypoxic Signature followed by reverse transcription-quantitative PCR Validation in Cancer Cell Lines
Source: Iran Biomed J. 2022 Dec 12;27(1):23–33. doi: 10.52547/ibj.3803 (PMC9971715; doi:10.52547/ibj.3803)
Supplement: Supplementary file 1 — Supplementary Fig. 1 [file ibj-27-23-s1.pdf]

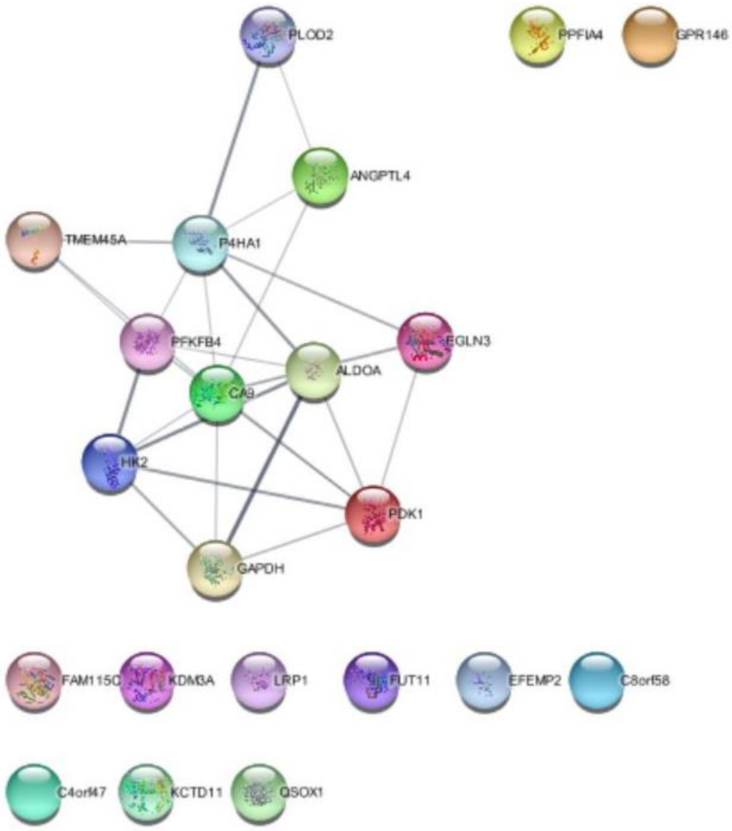

Supplementary Fig. 1. Protein-protein interaction network of common genes based on STRING database. To visualize the nodes, cytoscape spring-embedded layout algorithm was used.
